# Heme oxygenase-1 deficiency exacerbates angiotensin II-induced aortic aneurysm in mice

**DOI:** 10.18632/oncotarget.11917

**Published:** 2016-09-08

**Authors:** Yen-Chun Ho, Meng-Ling Wu, Pei-Yu Gung, Chung-Huang Chen, Cheng-Chin Kuo, Shaw-Fang Yet

**Affiliations:** ^1^ Institute of Cellular and System Medicine, National Health Research Institutes, Zhunan, Taiwan; ^2^ Graduate Institute of Life Sciences, National Defense Medical Center, Taipei, Taiwan; ^3^ Graduate Institute of Basic Medical Science and Metabolomic Research Center, China Medical University, Taichung, Taiwan

**Keywords:** aortic aneurysm, abdominal, heme oxygenase-1, oxidative stress, inflammation, Pathology Section

## Abstract

Abdominal aortic aneurysm (AAA) is a chronic but often fatal disease in elderly population. Heme oxygenase-1 (HO-1) is a stress response protein with antioxidative and anti-inflammatory properties. HO-1 has been shown to protect against atherogenesis and arterial intimal thickening. Emerging evidences suggest that AAA and arterial occlusive disease have distinct pathogenic mechanisms. Thus, in this study we investigated the role of HO-1 in angiotensin II-induced AAA formation in HO-1^+/+^apoE^−/−^ and HO-1^−/−^apoE^−/−^ mice. We found that complete loss of HO-1 increased AAA incidence and rupture rate, and drastically increased aneurysmal area and severity, accompanied with severe elastin degradation and medial degeneration. Interestingly, we often observed not only AAA but also thoracic aortic aneurysm in HO-1^−/−^apoE^−/−^ mice. Furthermore, reactive oxygen species levels, vascular smooth muscle cell (VSMC) loss, macrophage infiltration, matrix metalloproteinase (MMP) activity were markedly enhanced in the aneurysmal aortic wall in HO-1^−/−^apoE^−/−^ mice. In addition, HO-1^−/−^apoE^−/−^ VSMCs were more susceptible to oxidant-induced cell death and macrophages from HO-1^−/−^apoE^−/−^ mice had aggravated responses to angiotensin II with substantial increases in inflammatory cytokine productions and MMP9 activity. Taken together, our results demonstrate the essential roles of HO-1 in suppressing the pathogenesis of AAA. Targeting HO-1 might be a promising therapeutic strategy for AAA.

## INTRODUCTION

Abdominal aortic aneurysm (AAA) is a chronic but often fatal vascular disease that primarily affects older male patients and estimated to be the tenth commonest cause of mortality, responsible for ∼2% of all deaths [1]. Despite that mortality of other types of cardiovascular diseases has decreased, the rising age-standardized mortality of AAA indicates a rise in AAA incidence [1, 2]. AAA is a localized dilatation of the abdominal aorta exceeding the normal diameter by more than 50% [3], characterized by chronic aortic wall inflammation, loss of medial vascular smooth muscle cells (VSMCs), and connective tissue degradation and remodeling. Although the pathogenesis of AAA remains incompletely understood, it is well accepted that inflammation and oxidative stress are key factors inducing AAA formation [4, 5]. Inflammation-mediated proteolysis and excessive extracellular matrix breakdown of the aortic wall result in aortic expansion and aneurysm formation, and eventually rupture [6]. Reactive oxygen species (ROS) levels are markedly increased within human AAA segments compared with adjacent non-aneurysmal aortas [7] and in the aortic walls of experimental animals [5], implicating a critical role of ROS in AAA formation. Thus, reducing ROS generation and inflammation might be useful therapeutic strategies [8, 9].

Heme oxygenase-1 (HO-1) is a stress response protein and catalyzes the oxidation of heme to generate carbon monoxide (CO), biliverdin, and iron. These reaction products of HO-1 have potent anti-inflammatory and antioxidative functions [10]. Although HO-1 is expressed at low levels in most tissues under normal physiological conditions, it is highly inducible in response to various pathological stresses and serves as an adaptive defense mechanism to protect cells and tissues against injury in many disease settings. Despite a protective role of HO-1 in occlusive vascular diseases is well established [9], direct evidence is lacking about the role of HO-1 in AAA formation. In particular, recent evidences support a concept of separate developmental mechanisms underlying the pathogenesis of AAA and arterial occlusive diseases [11, 12]. In a rat porcine pancreatic elastase (PPE)-induced AAA model, flow loading attenuates AAA enlargement and also increases macrophage antioxidative gene expressions [13]. cDNA microarray analysis of elastase-induced rat AAA reveals that during AAA development genes involved in oxidative stress are upregulated [14]. Genetic polymorphism study suggests that HO-1 gene promoter (GT)_n_ repeats polymorphism that modulates HO-1 expression might be associated with AAA development in humans [15]. However, a different study reports no correlation between HO-1 (GT)_n_ repeats promoter polymorphism and AAA formation and progression in Croatian patients [16]. Despite the controversy, given the anti-inflammatory and antioxidative properties of HO-1, we hypothesized that HO-1 might play a protective role in the pathogenesis of AAA.

In the present study, we took a loss-of-function approach by first generating HO-1^−/−^apoE^−/−^ mice and then used these mice to test the role of HO-1 in the pathogenesis of angiotensin II-induced AAA. We demonstrate that HO-1 is induced in the aortic wall in response to angiotensin II infusion, possibly serving as an adaptive defense mechanism. A complete loss of HO-1 exacerbates aortic aneurysm formation *via* enhanced oxidative stress, inflammation, and matrix metalloproteinase (MMP) expression and activity.

## RESULTS

### HO-1 is induced in the aneurysmal segment during development of AAA

**Figure 1:**
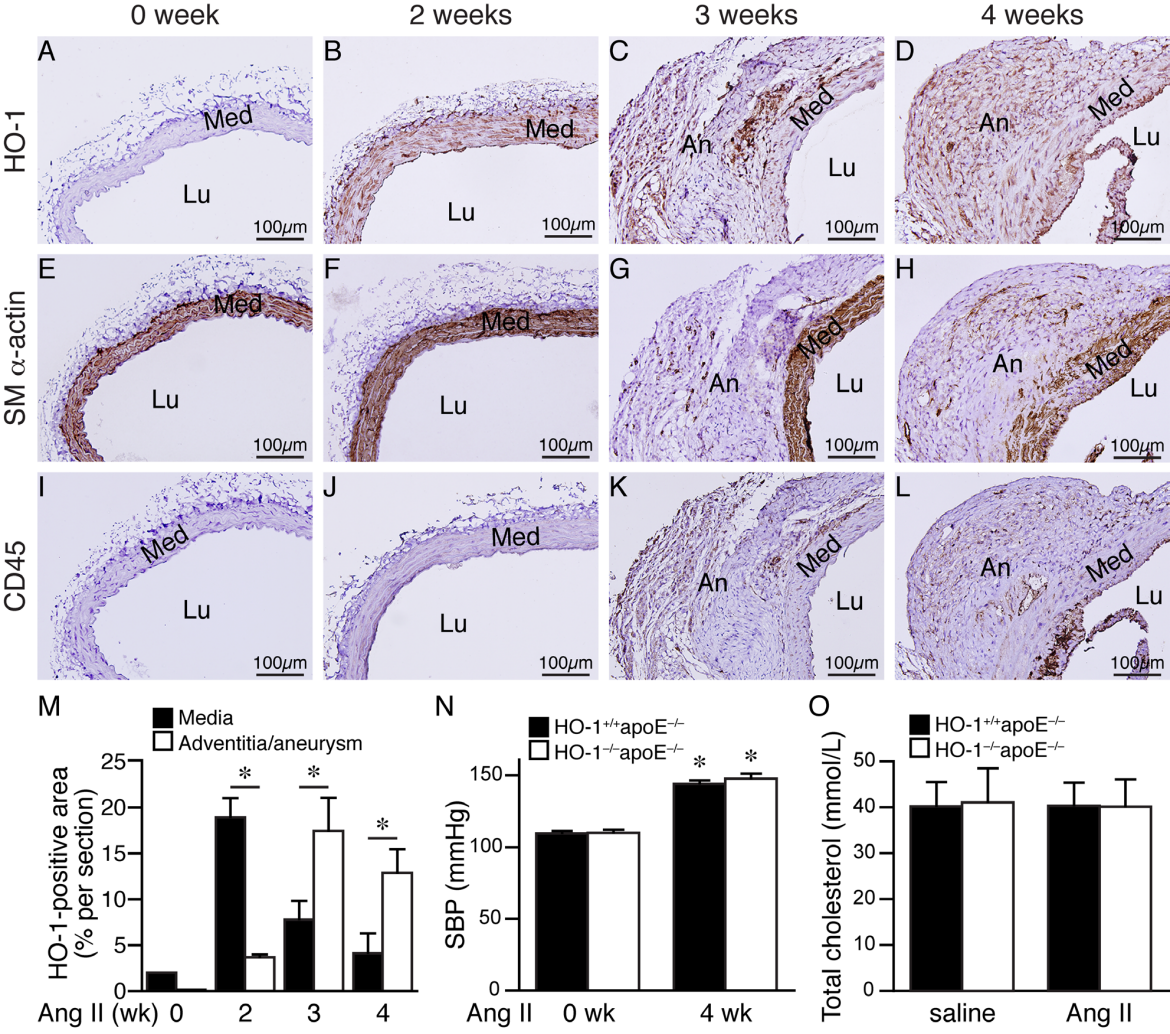
HO-1 induction in the aorta during AAA development HO-1^+/+^apoE^−/−^ mice were subjected to angiotensin II (Ang II) infusion and abdominal aortas harvested for immunohistochemistry to detect expressions (brown color) of HO-1 **A.**-**D.**, SM α-actin **E.**-**H.**, and CD45 **I.**-**L.** at 0 week **A.**, **E.**, and **I.**, 2 weeks **B.**, **F.**, and **J.**, 3 weeks **C.**, **G.**, and **K.**, and 4 weeks **D.**, **H.**, and **L.** following infusion (*n* = 3 each). Med, media; Lu, lumen; An, aneurysm. **M.** Expression levels of HO-1 in the media and adventitia/aneurysm were quantified and expressed as % per section. *P < 0.05. (*n* = 5, 6, 5, and 5 for 0, 2, 3, and 4 weeks, respectively). **N.** Systolic blood pressure (SBP) of mice before and after 4 weeks of angiotensin II infusion (*n* = 3 and 7 for HO-1^+/+^apoE^−/−^ and HO-1^−/−^apoE^−/−^, respectively). *P < 0.05 *vs*. 0 week in the same group. **O.** Total cholesterol levels from plasma of HO-1^+/+^apoE^−/−^ and HO-1^−/−^apoE^−/−^ mice infused with saline (*n* = 4 and 3, respectively) or angiotensin II (*n* = 5 and 4, respectively) and fed a high-fat diet for 4 weeks.

To investigate whether HO-1 has a role in AAA formation, we examined its temporal expression patterns in the abdominal aorta during the course of AAA development. Twelve-week-old HO-1^+/+^apoE^−/−^ mice were infused with angiotensin II and fed a high-fat diet to induce AAA. Aortas were harvested at 0, 2, 3, and 4 weeks later for histological analysis. Immunostaining of abdominal aortic tissues revealed that HO-1 was barely detectable before angiotensin II infusion (Figure 1A), consistent with the previous findings that HO-1 is expressed at very low levels in the aorta under normal physiological conditions [17]. Interestingly, HO-1 was substantially induced in the media 2 weeks later (Figure 1B). At 3 and 4 weeks, we observed HO-1 expression in the media and aneurysm/adventitia (Figure 1C and 1D). Quantitative analysis of HO-1-positive area at different time points after angiotensin II infusion revealed that HO-1 expression in the media was highest at 2 weeks while expression in the adventitia was lowest at 2 weeks (Figure 1M). HO-1 level subsequently decreased in the media at 3 and 4 weeks; in contrast, HO-1 level in the adventitia/aneurysm peaked at 3 weeks and maintained at high level at 4 weeks (Figure 1M). To determine cell types that expressed HO-1, we stained adjacent sections with smooth muscle (SM) α-actin and CD45 antibodies to identify VSMCs and immune cells, respectively. Indeed, immunohistochemistry showed that at 2 weeks, HO-1 was expressed in medial layer (Figure 1B and 1F) whereas no CD45-positive immune cells were detected in the vessel wall (Figure 1J). At 3 and 4 weeks, we observed expression of SM α-actin and CD45 in the aneurysm/adventitia, suggesting presence of myofibroblasts and immune cells (Figure 1G-1H and 1K-1L). This pattern of HO-1 expression suggests that it might be the medial VSMCs that first respond to angiotensin II, followed by infiltrated immune cells and fibroblasts in the adventitia/aneurysm. Our results indicate a potential important role of HO-1 in the pathogenesis of AAA.

### HO-1 deficiency aggravates angiotensin II-induced aortic aneurysm formation

To assess the role of HO-1 in AAA formation, we crossed apoE^−/−^ with HO-1^−/−^ mice [18] and subsequently generated HO-1^+/+^apoE^−/−^ and HO-1^−/−^apoE^−/−^ mice for the experimental AAA model. Although angiotensin II infusion significantly increased systolic blood pressure 4 weeks later compared with baseline level but there was no difference between the 2 groups of mice (Figure 1N). High-fat diet elevated plasma cholesterol to similar levels in both saline and angiotensin II groups and in HO-1^+/+^apoE^−/−^ and HO-1^−/−^apoE^−/−^ mice (Figure 1O).

**Figure 2:**
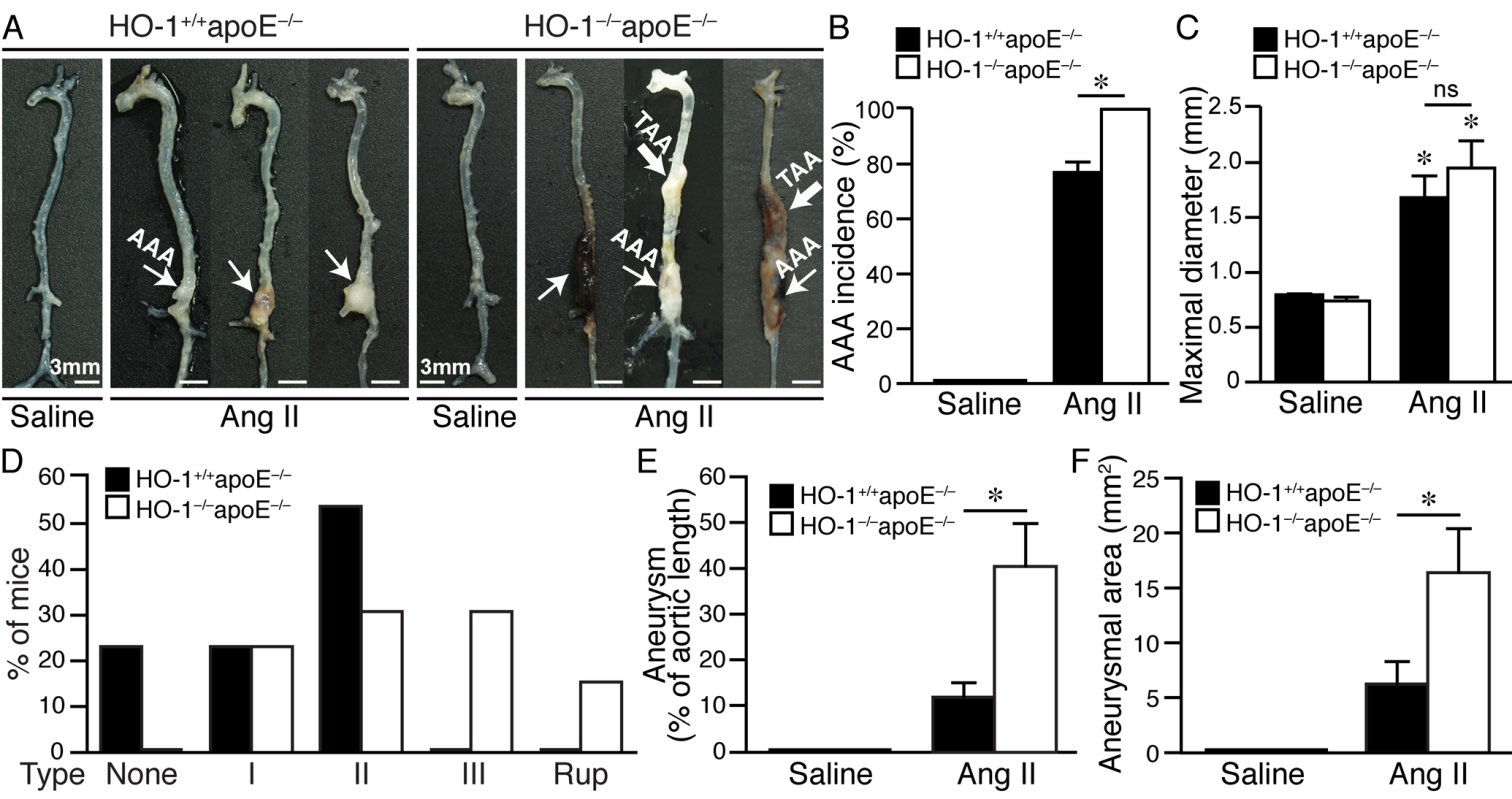
Absence of HO-1 exacerbates angiotensin II-induced aortic aneurysm formation in mice Mice were infused with saline or angiotensin II for 4 weeks. **A.** Representative aortas of HO-1^+/+^apoE^−/−^ mice infused with saline (*n* = 4) or angiotensin II (*n* = 13) and HO-1^−/−^apoE^−/−^ mice infused with saline (*n* = 5) or angiotensin II (*n* = 13) are shown. Thin arrow indicates abdominal aortic aneurysm (AAA) while thick arrow indicates thoracic aortic aneurysm (TAA). **B.** AAA incidence. *P < 0.05 *vs*. HO-1^+/+^apoE^−/−^ mice. **C.** Maximal diameter of suprarenal aorta. *P < 0.05 *vs*. saline; ns, no significance. **D.** Severity grade of AAA. Rup, rupture. **E.** Aneurysm was evaluated as % of aortic length. *P < 0.05 *vs*. HO-1^+/+^apoE^−/−^ mice. **F.** Aneurysm is quantified by measuring two-dimensional area (mm^2^). *P < 0.05 vs. HO-1^+/+^apoE^−/−^ mice.

**Figure 3:**
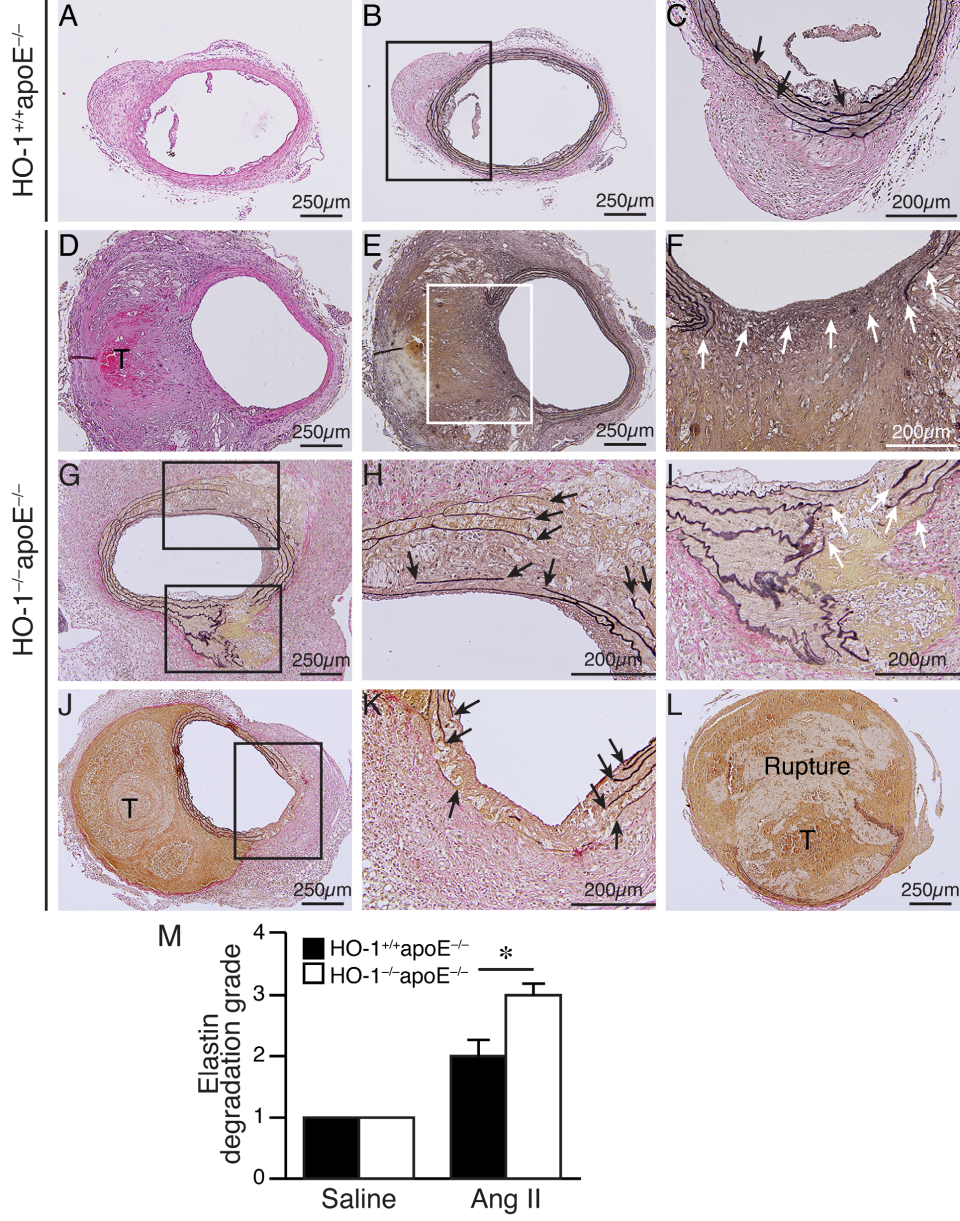
Enhanced elastin degradation in angiotensin II-infused HO-1^−/−^apoE^−/−^ mice HO-1^+/+^apoE^−/−^ **A.**-**C.** and HO-1^−/−^apoE^−/−^ **D.**-**L.** mice were infused with angiotensin II for 4 weeks and abdominal aortas harvested for histological analysis. H&E **A.** and **D.** and Verhoeff's elastin staining **B.**-**C.** and **E.**-**L.** of mouse AAA sections. **C.** and **F.** Higher magnification of the boxed areas in **B.** and **E.**, respectively. **H.** and **I.** Higher magnification of the boxed areas in G. **K.** Higher magnification of the boxed area in J. Arrows indicate disrupted elastin fibers. T, thrombus. **L.** A ruptured AAA. **M.** Quantitative analysis of elastin degradation grade from HO-1^+/+^apoE^−/−^ mice infused with saline (*n* = 4) or angiotensin II (*n* = 13) and HO-1^−/−^apoE^−/−^ mice infused with saline (*n* = 5) or angiotensin II (*n* = 13). *P < 0.05 *vs*. HO-1^+/+^apoE^−/−^ mice.

Saline infusion did not cause any aneurysm formation (Figure 2A-2C and 2E-2F). Angiotensin II induced AAA in 77% of HO-1^+/+^apoE^−/−^ and 100% in HO-1^−/−^apoE^−/−^ mice (Figure 2B, *P* < 0.05). Compared with saline, angiotensin II significantly increased aneurysm diameter; although the diameter was slightly larger in HO-1^−/−^apoE^−/−^ than HO-1^+/+^apoE^−/−^ mice it did not reach a statistical significance (Figure 2C). According to Daugherty's modified classification of AAA (none, I, II, III, and rupture) [19], most (54%) of HO-1^+/+^apoE^−/−^ mice exhibited type II aneurysm, 23% type I, 23% no aneurysm, and no type III or rupture observed. By contrast, lack of HO-1 markedly enhanced aneurysm severity, skewing toward more severe types: 23% type I, 31% type II, 31% type III, and a 15.4% rupture rate (Figure 2A and 2D). Further morphological analysis revealed that HO-1 deficiency significantly increased aneurysm along the aortic length from 12±3% to 41±9% (*P* < 0.05) and aneurysmal 2-dimensional area from 6±2 mm^2^ to 16±4 mm^2^ (*P* < 0.05) (Figure 2A, 2E, and 2F). Interestingly, we often observed not only AAA but also thoracic aortic aneurysm (TAA) in HO-1^−/−^apoE^−/−^ mice (46% *vs*. 0% HO-1^+/+^apoE^−/−^; *P* = 0.007 Fisher's exact test) (Figure 2A). Concomitant formation of AAA and TAA is not common in mouse aneurysm model and appears to be unique to HO-1^−/−^apoE^−/−^ mice. In addition, we also observed increased, although not significant (62% *vs*. 38% HO-1^+/+^apoE^−/−^; *P* = 0.11, Fisher's exact test), thrombus formation in HO-1^−/−^apoE^−/−^ mice (Figure 2A). Histological analysis with H&E and elastin staining showed normal morphology and elastin layers of suprarenal aorta in saline-treated HO-1^+/+^apoE^−/−^ and HO-1^−/−^apoE^−/−^ mice (data not shown). Angiotensin II infusion resulted in AAA formation (Figure 3A and 3D) and elastin layer breakage as revealed by Verhoeff's elastin stain (Figure 3B-3C and 3E-3L). Examination of HO-1^−/−^apoE^−/−^ mouse AAAs showed that many exhibited severe elastin degradation (Figure 3E, 3G, and 3J). The breakage was more evident at higher magnifications (Figure 3F, 3H-3I, and 3K). Quantitative analysis showed a higher elastin degradation grade in HO-1^−/−^apoE^−/−^ AAAs (3.0±0.2) compared with 2.0±0.3 of HO-1^+/+^apoE^−/−^AAAs (*P* < 0.05) (Figure 3M). Further examination of AAA sections revealed thrombus formation in many HO-1^−/−^apoE^−/−^ mice (Figure 3D, 3G, 3J, and 3L). Aortic dissection (Figure 3I) and rupture (Figure 3L) were also observed. These results demonstrate that a complete loss of HO-1 aggravates aortic aneurysm formation.

### Complete loss of HO-1 increases ROS levels and VSMC loss in angiotensin II-infused mouse aorta

**Figure 4:**
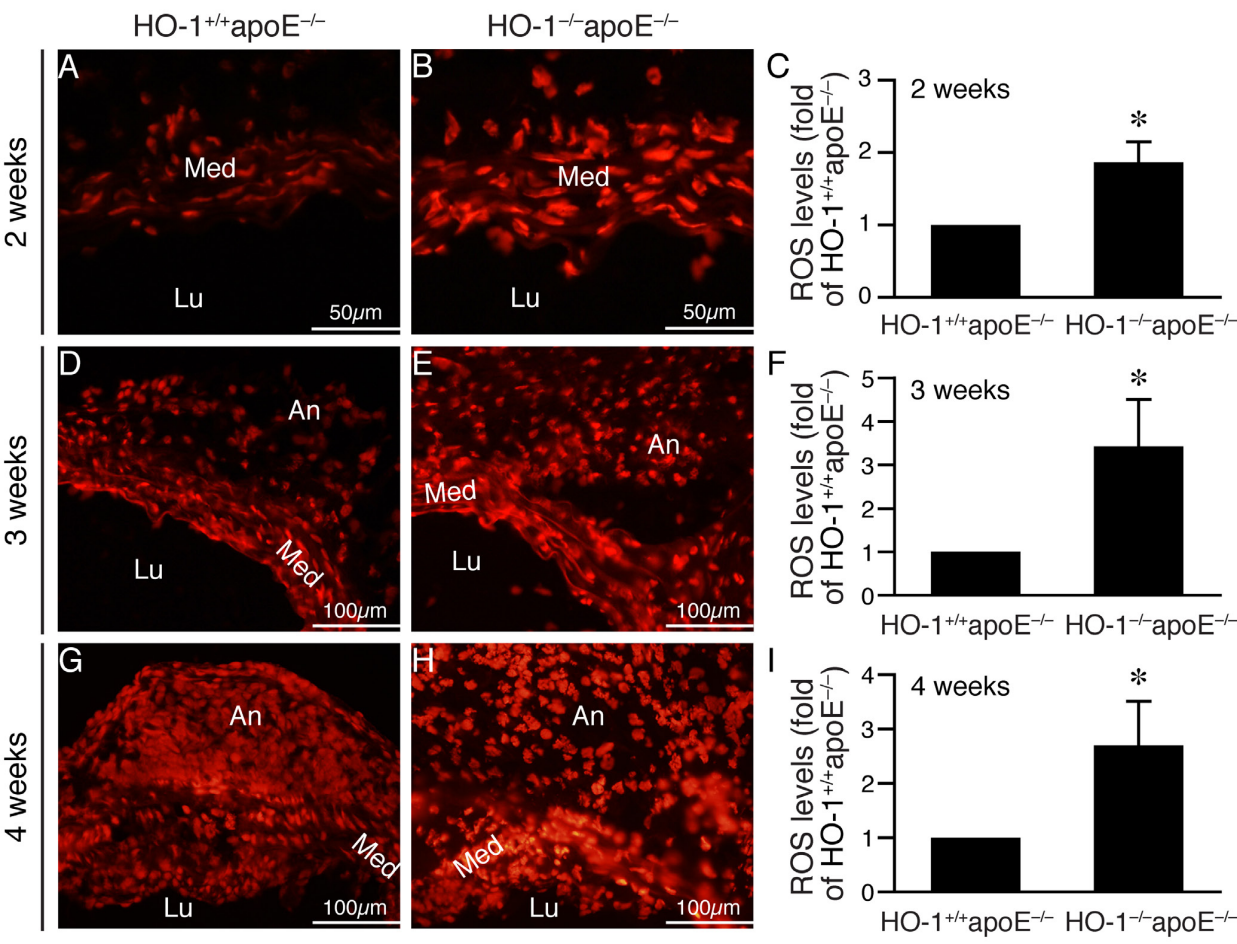
Lack of HO-1 enhances ROS levels in the aortic wall of angiotensin II-infused mice Mice were infused with angiotensin II and abdominal aortas harvested at different time points. DHE staining (red fluorescence) was performed to assess ROS levels. **A.**-**B.** Representative images of DHE staining from abdominal aneurysmal segment 2 weeks after angiotensin II infusion. **C.** Quantification of DHE staining 2 weeks after angiotensin II infusion from HO-1^+/+^apoE^−/−^ (*n* = 4) and HO-1^−/−^apoE^−/−^ (*n* = 5) mice and expressed as fold of HO-1^+/+^apoE^−/−^ mice. *P < 0.05 *vs*. HO-1^+/+^apoE^−/−^ mice. **D.**-**E.** Representative images of DHE staining from aneurysmal segment 3 weeks after angiotensin II infusion. **F.** DHE staining of aortic sections from HO-1^+/+^apoE^−/−^ (*n* = 4) and HO-1^−/−^apoE^−/−^ (*n* = 4) mice 3 weeks after angiotensin II infusion was quantified and expressed as fold of HO-1^+/+^apoE^−/−^ mice. *P < 0.05 *vs*. HO-1^+/+^apoE^−/−^ mice. **G.**-**H.** Representative images of DHE staining from aneurysmal segment 4 weeks after angiotensin II infusion. **I.** Quantification of DHE staining 4 weeks after angiotensin II infusion from HO-1^+/+^apoE^−/−^ (*n* = 4) and HO-1^−/−^apoE^−/−^ (*n* = 4) mice and expressed as fold of HO-1^+/+^apoE^−/−^ mice. *P < 0.05 *vs*. HO-1^+/+^apoE^−/−^ mice. Med, media; Lu, lumen; An, aneurysm.

**Figure 5:**
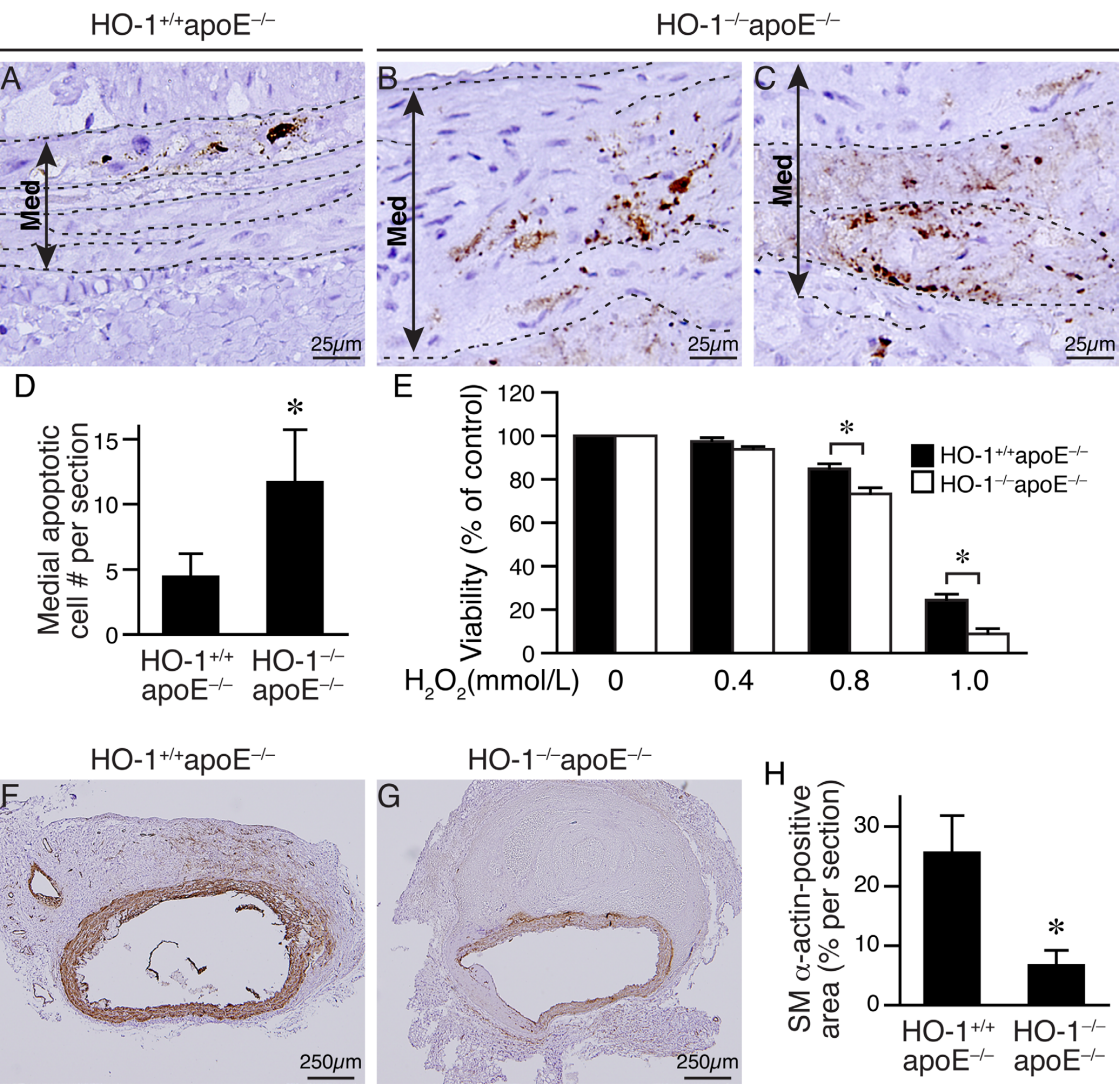
Loss of HO-1 increases VSMC death and reduces VSMC content in AAAs of angiotensin II-infused mice Mice were infused with angiotensin II for 4 weeks and AAAs harvested for histological analysis. **A.**-**C.** Apoptotic cells were identified by cleaved caspase-3 staining (brown color) in abdominal aortic aneurysmal sections of angiotensin II-infused HO-1^+/+^apoE^−/−^ (*n* = 9) and HO-1^−/−^apoE^−/−^ (*n* = 6) mice, respectively. Elastin layers are indicated with dashed lines. A double arrowed line demarcates medial layer (Med). Representative sections are shown. **D.** Quantitative analysis of cleaved caspase-3-positive cells in the medial layer. *P < 0.05 *vs*. HO-1^+/+^apoE^−/−^ mice. **E.** Primary HO-1^+/+^apoE^−/−^ and HO-1^−/−^apoE^−/−^ VSMCs (5 different isolates from each genotype) were treated with increasing concentrations of H_2_O_2_ and viability measured after 24 h. *P < 0.05 *vs*. HO-1^+/+^apoE^−/−^ VSMCs. **F.**-**G.** SM α-actin staining (brown) of aneurysmal aortic sections from HO-1^+/+^apoE^−/−^ (*n* = 6) and HO-1^−/−^apoE^−/−^ (*n* = 6) mice. **H.** SM α-actin contents of vessel sections were quantified and expressed as % per section. *P < 0.05 (HO-1^−/−^apoE^−/−^ *vs*. HO-1^+/+^apoE^−/−^ mice, *n* = 6 each).

Given that HO-1 has antioxidant capacity, we hypothesized that absence of HO-1 might result in enhanced ROS accumulation in the aortic wall after angiotensin II infusion. DHE staining to assess ROS levels showed more intense staining in the aortic wall, particularly in the medial layer of HO-1^−/−^apoE^−/−^ mice at 2 weeks (Figure 4A and 4B), 1.9±0.3 fold higher than that of HO-1^+/+^apoE^−/−^ mice (*P* < 0.05; Figure 4C). At 3 and 4 weeks, we observed strong DHE staining in the media and aneurysms in both genotypes of mice. In comparison, the staining intensity of HO-1^−/−^apoE^−/−^ mice was much stronger than HO-1^+/+^apoE^−/−^ mice at both time points (Figure 4D-4I). Since ROS can induce VSMC apoptosis/death [20], we evaluated medial apoptosis in the aneurysmal aorta. Immunostaining with cleaved caspase-3 antibody revealed more apoptotic cells in the aortic media from HO-1^−/−^apoE^−/−^ than HO-1^+/+^apoE^−/−^ mice (Figure 5A-5C, brown), with 11.8±4.0 *vs*. 4.5±1.7 apoptotic cells per section, respectively (Figure 5D, *P* < 0.05). Furthermore, HO-1^−/−^apoE^−/−^ VSMCs were more susceptible to oxidant-induced cell death than HO-1^+/+^apoE^−/−^ VSMCs (Figure 5E). Corroborating these results, lack of HO-1 reduced VSMC marker SM α-actin staining in the aneurysmal segments (Figure 5F and 5G), significantly decreased the positive area from 25.8±6.1% in HO-1^+/+^apoE^−/−^ to 6.9±2.4% in HO-1^−/−^apoE^−/−^ aorta (Figure 5H). These findings demonstrate that lack of HO-1 reduces VSMC content in the aneurysmal aorta.

### Loss of HO-1 enhances MMP activity and macrophage infiltration in angiotensin II-infused mouse aorta

**Figure 6:**
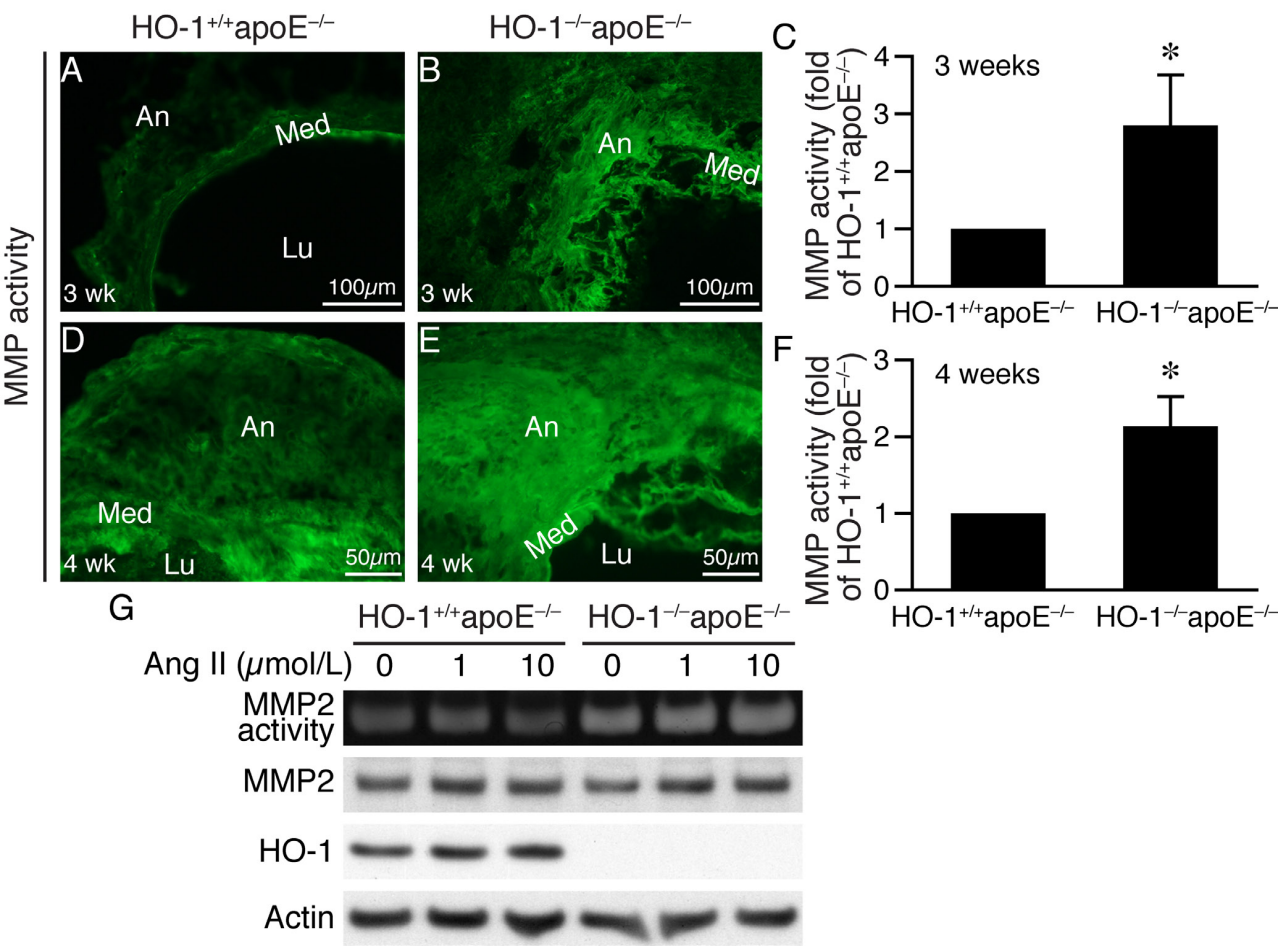
Lack of HO-1 enhances MMP activity in AAAs and VSMCs *In situ* zymography was performed to measure MMP activity (green fluorescence) on AAA sections from HO-1^+/+^apoE^−/−^ and HO-1^−/−^apoE^−/−^ mice. **A.**-**B.** Three weeks after angiotensin II infusion (*n* = 4 each group). **C.** MMP activity of 3 weeks was quantified and expressed as fold of HO-1^+/+^apoE^−/−^ mice. *P < 0.05 *vs*. HO-1^+/+^apoE^−/−^ mice. **D.**-**E.** Four weeks (*n* = 4 each genotype) after angiotensin II infusion. Med, media; Lu, lumen; An, aneurysm. **F.** MMP activity of 4 weeks was quantified and expressed as fold of HO-1^+/+^apoE^−/−^ mice. *P < 0.05 *vs*. HO-1^+/+^apoE^−/−^ mice. **G.** HO-1^+/+^apoE^−/−^ and HO-1^−/−^apoE^−/−^ VSMCs (*n* = 3 different isolates from each genotype) were stimulated with different concentrations of angiotensin II for 24 h and conditioned medium collected for zymography to examine MMP2 activity. Total proteins from cells were prepared for Western blotting for MMP2 and HO-1 expressions. Equivalent loading was verified by probing with a pan-actin antibody.

The more severe elastin degradation of HO-1^−/−^apoE^−/−^ mouse aneurysmal aorta (Figure 3) is likely attributed to MMP activity. We thus assessed MMP activity in the aortic wall by *in situ* zymography. Compared with HO-1^+/+^apoE^−/−^ mice, MMP activity (green fluorescence) was significantly enhanced 2-3-fold in HO-1^−/−^apoE^−/−^ mice at both 3 weeks (Figure 6A-6C) and 4 weeks (Figure 6D-6F) after angiotensin II infusion. To determine whether VSMCs contribute to MMP activity, we measured MMP2 activity and expressions in HO-1^+/+^apoE^−/−^ and HO-1^−/−^apoE^−/−^ VSMCs. Angiotensin II increased MMP2 activity in cells of both genotypes 24 h after stimulation. However, MMP2 activity was further enhanced in HO-1^−/−^apoE^−/−^ VSMCs, despite that angiotensin II increased MMP2 expression to similar levels in both genotypes (Figure 6G). Although the mechanisms are not clear regarding the discrepancy between extracellular MMP activity and intracellular MMP2 level, it might be that in VSMCs, active forms of MMP2 are efficiently secreted into conditioned medium. Interestingly, angiotensin II increased HO-1 expressions in HO-1^+/+^apoE^−/−^ VSMCs (Figure 6G).

**Figure 7:**
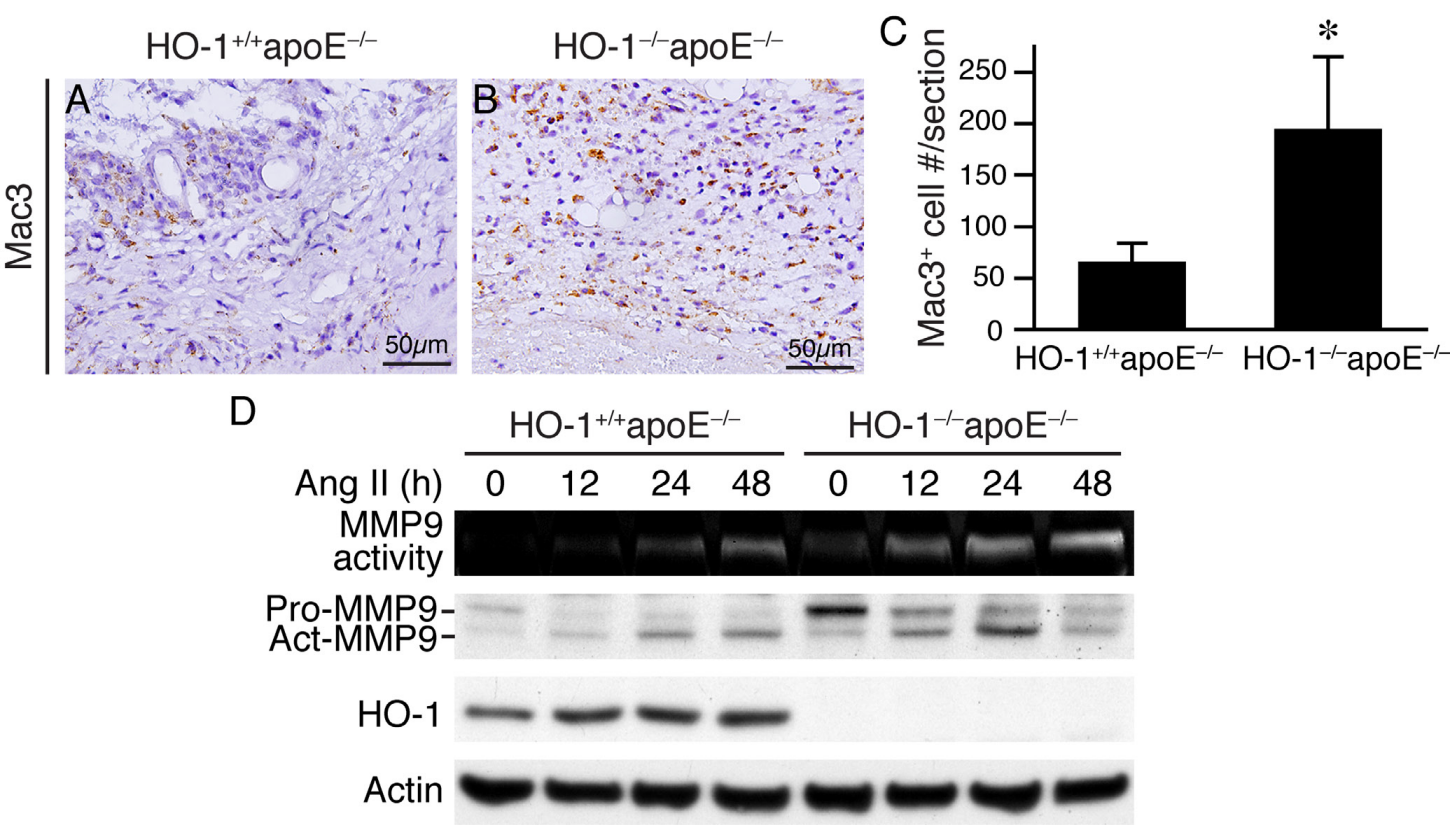
An absence of HO-1 enhances macrophage infiltration in AAAs and MMP9 activity in macrophages Mice were infused with angiotensin II for 4 weeks and AAAs harvested for histological analysis. **A.**-**B.** Mac3 immunostaining (brown color) was performed on HO-1^+/+^apoE^−/−^ and HO-1^−/−^apoE^−/−^ (*n* = 13 and 10, respectively) mouse aortic sections to identify macrophages. **C.** Quantification of infiltrated Mac3^+^ cells and expressed as Mac3^+^ cells per section. *P < 0.05 *vs*. HO-1^+/+^apoE^−/−^ mice. **D.** Peritoneal macrophages from HO-1^+/+^apoE^−/−^ and HO-1^−/−^apoE^−/−^ mice (*n* = 3 different isolates from each genotype) were stimulated with angiotensin II (10 μmol/L). Conditioned medium and cell lysates were harvested at different time points for zymography to measure MMP9 activity and Western blotting to detect MMP9 and HO-1 expressions, and actin for normalization.

The results that MMP active area extended beyond medial layer (Figure 6A-6B and 6D-6E) indicated that cells other than VSMCs might also contribute to MMP activity in the aneurysmal aortic wall. Since inflammation is implicated in AAA development [21], we first investigated inflammatory cell infiltration into the aortic wall. Indeed, immunostaining revealed a 3-fold increase of Mac3-positive cells in HO-1^−/−^apoE^−/−^ than HO-1^+/+^apoE^−/−^ mouse AAA (Figure 7A-7C), indicating increased macrophage infiltration. Furthermore, zymography assays showed that in primary macrophages angiotensin II increased MMP9 activity in a time-dependent manner and lack of HO-1 further enhanced the activity (Figure 7D). Western blotting showed that angiotensin II increased both proform and active form of MMP9 to higher levels in HO-1^−/−^apoE^−/−^ macrophages (Figure 7D). As in VSMCs, angiotensin II induced HO-1 protein expressions in HO-1^+/+^apoE^−/−^ macrophages (Figure 7D). Together these data indicate that loss of HO-1 increases macrophage infiltration into the aortic wall, enhances MMP activity in VMSCs and macrophages, resulting in increased elastin layer degradation.

### Lack of HO-1 aggravates inflammatory responses in AAAs and in angiotensin II-treated macrophages

**Figure 8:**
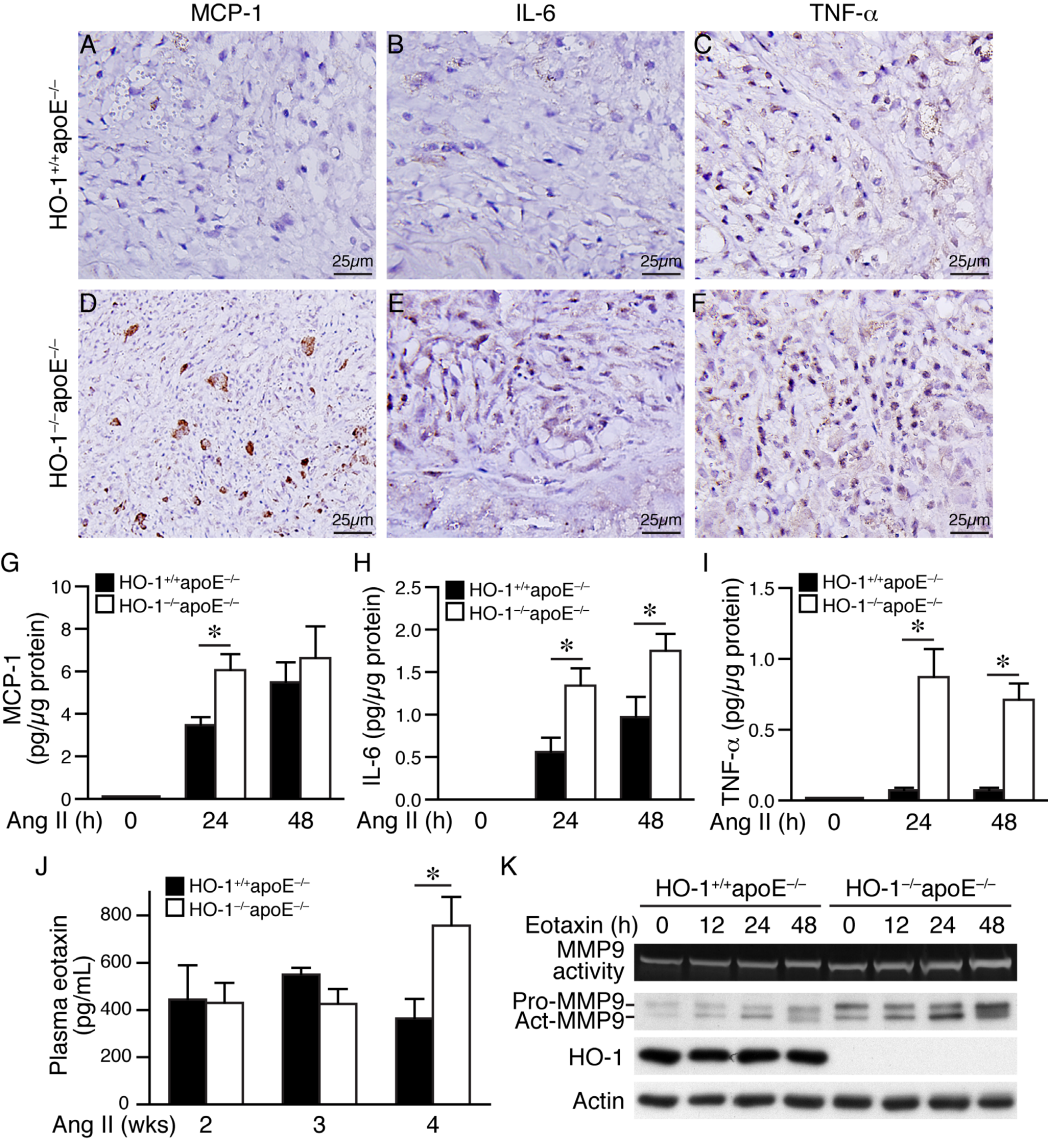
Lack of HO-1 aggravates inflammatory responses in AAAs and in angiotensin II-treated macrophages **A.**-**F.** Four weeks after angiotensin II infusion, HO-1^+/+^apoE^−/−^ and HO-1^−/−^apoE^−/−^ mouse AAA sections were immunostained with antibodies for MCP-1 **A.** and **D.**, IL-6 **B.** and **E.**, or TNF-α **C.** and **F.** Brown color indicates positive staining. **G.**-**I.** Peritoneal macrophages from HO-1^+/+^apoE^−/−^ and HO-1^−/−^apoE^−/−^ mice (3-4 different isolates from each genotype) were stimulated with angiotensin II (10 μmol/L) and conditioned medium harvested at different time points. Concentrations of inflammatory cytokines were measured using an ELISA kit, normalized to total protein amount and expressed as pg/μg protein. **G.** MCP-1 (*n* = 4 each genotype). **H.** IL-6 (*n* = 3 each genotype). **I.** TNF-α (*n* = 4 each genotype). Values are mean ± SE. *P < 0.05 *vs*. HO-1^+/+^apoE^−/−^ macrophages. **J.** Blood samples from HO-1^+/+^apoE^−/−^ and HO-1^−/−^apoE^−/−^ mice 2 weeks (*n* = 3 and 4, respectively), 3 weeks (*n* = 7 and 4, respectively), and 4 weeks (*n* = 9 and 11, respectively) following angiotensin II infusion were collected and plasma concentrations of eotaxin measured. *P < 0.05 *vs*. HO-1^+/+^apoE^−/−^ mice. **K.** HO-1^+/+^apoE^−/−^ and HO-1^−/−^apoE^−/−^ peritoneal macrophages (*n* = 3 different isolates from each genotype) were stimulated with eotaxin (100 ng/mL). Conditioned medium and cell lysates were harvested at different time points for zymography (MMP9 activity) and Western blotting to detect MMP9 and HO-1 expressions, and actin for normalization.

To further investigate the effects of HO-1 on inflammatory responses, we performed immunostaining on AAA sections using antibodies for monocyte chemoattractant protein-1 (MCP-1), IL-6, and TNF-α. Immunohistochemistry revealed increased expressions of these cytokines in HO-1^−/−^apoE^−/−^ AAAs when compared with that of HO-1^+/+^apoE^−/−^ AAAs (Figure 8A-8F). To further confirm these findings, we treated peritoneal macrophages with angiotensin II and measured inflammatory cytokine productions. ELISA assays showed that MCP-1, IL-6, and TNF-α were barely detectable before stimulation while angiotensin II substantially elevated the productions of these cytokines in both genotypes (Figure 8G-8I). Importantly, the levels of these inflammatory cytokines were further elevated in HO-1^−/−^apoE^−/−^ than HO-1^+/+^apoE^−/−^ macrophages (Figure 8G-8I).

### Four weeks angiotensin II infusion elevates plasma eotaxin levels in HO-1^−/−^apoE^−/−^ mice

A previous study showed that chemokine eotaxin is increased in the plasma of AAA patients [22] and another study found high concentration of eotaxin in the lumen of human cerebral aneurysms [23]. A recent study suggested eotaxin to be an independent plasma biomarker for AAA [24]. Moreover, eotaxin has been reported to induce pro-MMP2 expressions in VSMCs [25]. These collective findings implicate a role of eotaxin in aneurysm formation. Therefore, we measured eotaxin plasma concentration in mice after angiotensin II infusion. Eotaxin level was similar between HO-1^+/+^apoE^−/−^ and HO-1^−/−^apoE^−/−^ mice at 2 and 3 weeks (Figure 8J). Interestingly, plasma eotaxin was significantly elevated in HO-1^−/−^apoE^−/−^ than HO-1^+/+^apoE^−/−^ mice at 4 weeks (758±121 *vs*. 364±83 pg/mL, respectively) (Figure 8J), suggesting eotaxin might be produced at a later stage during AAA progression. Zymography revealed that eotaxin increased macrophage MMP9 activity and expressions, both of which were further enhanced in the absence of HO-1 (Figure 8K), suggesting an important role of HO-1 in controlling eotaxin level and the subsequent MMP activity at a later stage during AAA development.

### CO-releasing molecule CORM-3 abrogates angiotensin II-induced inflammatory cytokine productions in HO-1^−/−^apoE^−/−^ macrophages

**Figure 9:**
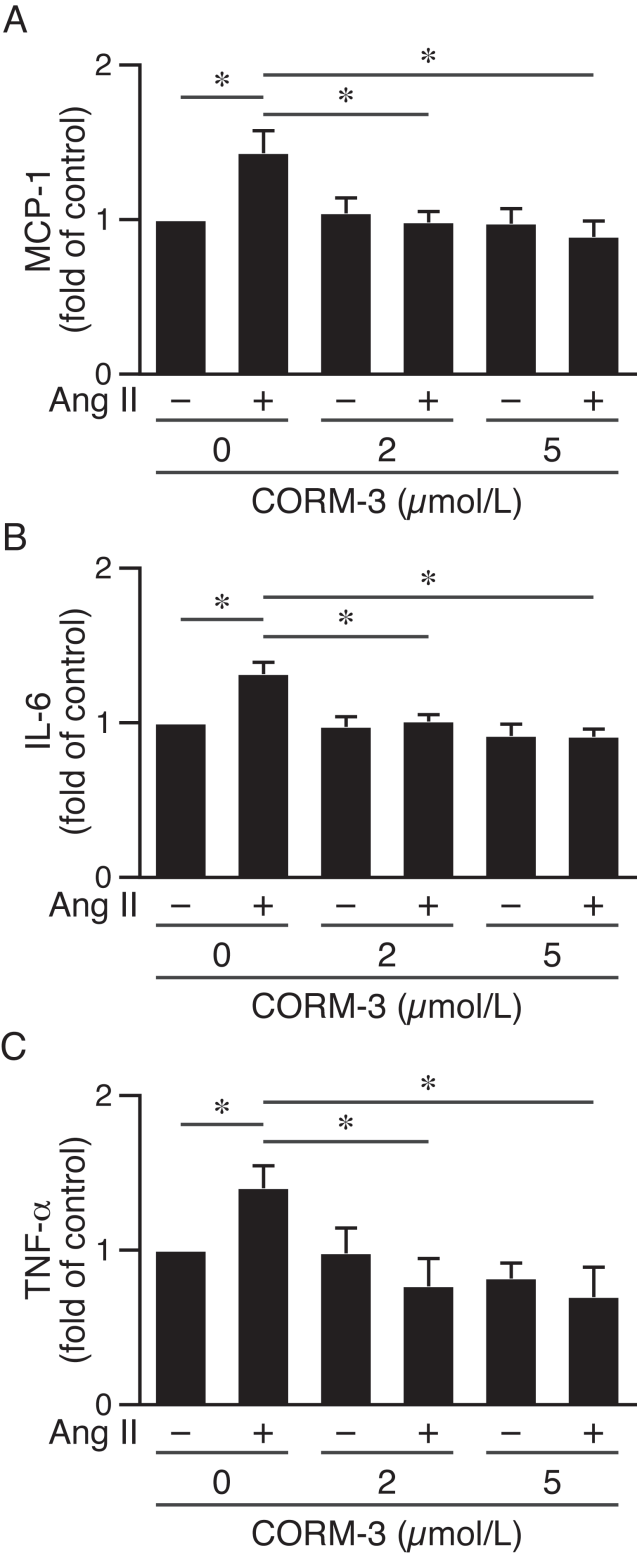
CORM-3 reduces angiotensin II-induced inflammatory cytokine productions in HO-1^−/−^apoE^−/−^ macrophages Peritoneal macrophages from HO-1^−/−^apoE^−/−^ mice were harvested (4 different isolates) and treated with 0, 2 or 5 μmol/L CORM-3 in the presence or absence of angiotensin II (10 μmol/L). Conditioned medium were harvested 24 h later. Inflammatory cytokines were measured using ELISA kits, normalized to total protein amount and expressed as fold of control. **A.** MCP-1 (*n* = 4). **B.** IL-6 (*n* = 4). **C.** TNF-α (*n* = 4). Values are mean ± SE. *P < 0.05 *vs*. macrophages treated with angiotensin II but without CORM-3.

To further confirm HO-1′s protective role, we performed rescue experiments by treating HO-1^−/−^apoE^−/−^ macrophages with angiotensin II and a CO-releasing molecule CORM-3, and then assessed inflammatory cytokine productions. CORM-3 suppressed angiotensin II-induced inflammatory cytokines MCP-1, IL-6, and TNF-α (Figure 9), indicating CORM-3 rescued the cellular deficits of HO-1^−/−^apoE^−/−^ macrophages and a protective role of HO-1.

## DISCUSSION

In this study, we examined the role of HO-1 in AAA using a loss-of-function approach in an angiotensin II-infused animal model. A complete loss of HO-1 enhances oxidative stress, medial VSMC loss, MMP activity, and macrophage infiltration in the aortic wall, leading to exacerbated abdominal and thoracic aortic aneurysm formation. We unequivocally demonstrate a crucial role of HO-1 in the pathogenesis of aortic aneurysm.

Our data indicate that a complete absence of the inducible stress response gene HO-1 leads to severe exacerbation of aortic aneurysm and increased rupture rate, which is a detrimental clinical consequence. Importantly, in addition to AAA, we frequently observed TAA in HO-1^−/−^apoE^−/−^ mice. Given that TAA is not common in mouse aneurysm model, this novel finding further demonstrates a critical role of HO-1 in the pathogenesis of aortic aneurysm. Concomitant with exacerbated AAA, ROS levels and medial VSMC apoptosis were markedly increased in the aortic wall of HO-1^−/−^apoE^−/−^ mice, supporting an antioxidative and anti-apoptotic role of HO-1 [26]. Moreover, HO-1^−/−^apoE^−/−^ VSMCs were more susceptible to oxidant-induced cell death than HO-1^+/+^apoE^−/−^ cells. As such, it is not surprising that HO-1 deficiency reduced VSMC content in the aneurysmal aorta (Figure 5E-5G). In addition to inducing VSMC death, ROS have been reported to activate MMP2 in VSMCs [27]. Indeed, we found that an absence of HO-1 further enhanced angiotensin II-induced MMP2 activity, which conceivably could contribute to increased elastin degradation and medial degeneration. Given that VSMCs are the major source for extracellular matrix productions, our findings support the view that medial VSMC death contributes to the reduction of cellularity and subsequent impairment for the repair and maintenance of the aortic extracellular matrix in AAAs [28, 29].

As macrophage infiltration into the aneurysmal aortic wall is a hallmark of AAA pathology, we examined inflammatory responses during AAA formation. We observed enhanced macrophage infiltration into the aortic wall of HO-1^−/−^apoE^−/−^ compared with HO-1^+/+^apoE^−/−^ mice. Macrophages from HO-1^−/−^apoE^−/−^ mice produced larger amounts of inflammatory cytokines MCP-1, IL-6, and TNF-α after angiotensin II stimulation (Figure 8A-8I); these cytokines could in turn act on VSMCs and macrophages to perpetuate vicious cycles of inflammation, ultimately resulting in exaggerated inflammatory response in the absence of HO-1. HO-1 has been implicated in IL-10-mediated MMP9 reduction in macrophages [30]. In response to angiotensin II, absence of HO-1 significantly enhances MMP9 activity in primary macrophages (Figure 7D), providing direct evidence of HO-1 in regulating MMP9, a major MMP in macrophages. Our findings that upon angiotensin II stimulation, lack of HO-1 increases MMP2 in VSMCs and MMP9 in macrophages are in line with a previous study suggesting macrophage-derived MMP9 and mesenchymal cell MMP2 are both required and work in concert to produce AAA [31]. The effects of HO-1 on MMPs may influence the development and stability of AAAs by attenuating elastin degradation and thereby preventing AAA rupture.

Interestingly, eotaxin is elevated in the plasma of angiotensin II-infused HO-1^−/−^apoE^−/−^ mice at 4 but not 2 or 3 weeks, suggesting lack of HO-1 increases eotaxin production at a later stage of AAA development, although the mechanisms by which HO-1 controls eotaxin expression remain to be determined. Importantly, eotaxin enhances MMP9 activity in HO-1^−/−^apoE^−/−^ in comparison with HO-1^+/+^apoE^−/−^ macrophages (Figure 8K). Of note, immunohistochemical analysis of human atherosclerosis revealed that eotaxin is predominantly located in SMCs while its receptor CCR3 in macrophage-rich regions [32]. Further, TNF-α treatment of human aortic SMCs markedly induces eotaxin expression [32]. Recently, eotaxin level (as assessed by qPCR) was found to be elevated within AAA adventitial tissues [24]. Taken together, it is likely that VSMCs and adventitial fibroblasts might upregulate eotaxin, which then act upon macrophages to enhance MMP9 activity. Collectively, these data unequivocally demonstrate that enhanced macrophage infiltration and elevated MMP9 activity contribute substantially to the extensive MMP activity and aortic wall degeneration in HO-1^−/−^apoE^−/−^ mouse aneurysmal segment. These findings are consistent with the notion that HO-1 induction might serve as an adaptive protective mechanism to limit macrophage infiltration and MMP activity.

Supporting our findings, a recent study using FVB wild type and heterozygous HO-1 (HO-1^+/−^) mice in a PPE-induced AAA model showed that HO-1^+/−^ mice have enhanced macrophage infiltration in AAA [33]. PPE model is characterized by destruction of elastic tissues followed by influx of inflammatory cells [34]. In contrast to increased AAA diameter (measured by ultrasound) in HO-1^+/−^ mice reported by Azuma et al, we found no significant difference of maximal aortic diameter between angiotensin II-infused HO-1^+/+^apoE^−/−^ and HO-1^−/−^apoE^−/−^ mice. This discrepancy might be due to different model and different type of mice used. Instead of a reduced level of HO-1 in HO-1^+/−^ mice, HO-1^−/−^apoE^−/−^ mice are completely devoid of HO-1 expression. Further, the pathological features of angiotensin II-induced AAA more mimic hallmarks of human AAA pathology [34]. Although aortic diameter was not significantly increased, HO-1^−/−^apoE^−/−^ mice exhibited increased area of aneurysm, exacerbated pathological features including enhanced elastin degradation, higher oxidative stress levels, increased medial VSMC loss, and enhanced MMP activity. More importantly, we found that 46% of HO-1^−/−^apoE^−/−^ mice developed not only AAA but also TAA, a feature unique to HO-1^−/−^apoE^−/−^ mice and would be difficult to detect using PPE model. Interestingly, low doses of the HMG-CoA reductase inhibitor rosuvastatin can induce HO-1 expression in aortic tissue and suppress AAA progression in the absence of lipid lowering [33]. Collectively, these findings emphasize a crucial role of HO-1 in aortic aneurysm formation.

**Figure 10:**
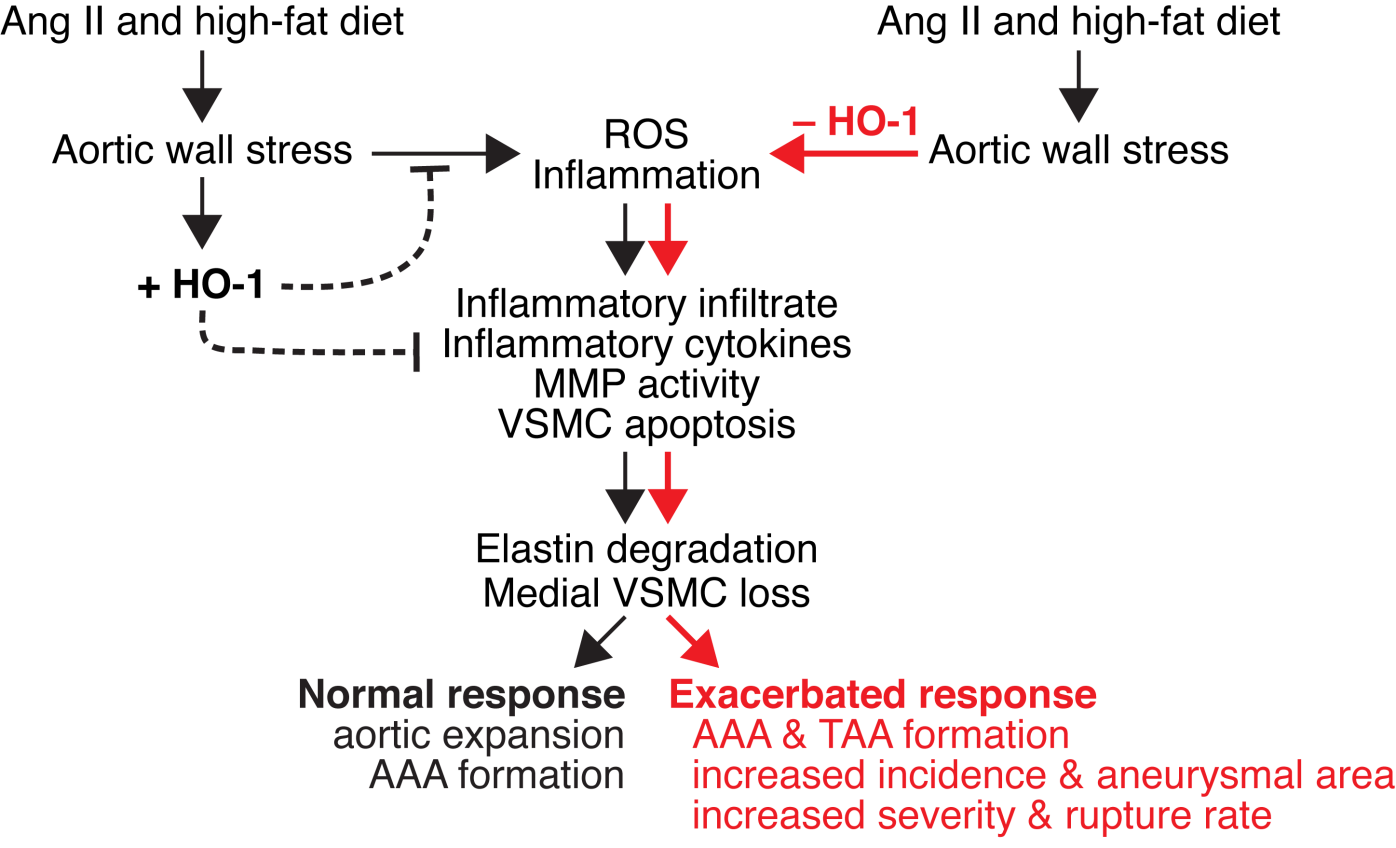
Schematic model of effect of HO-1 on angiotensin II-induced aortic aneurysm formation Angiotensin II and high-fat diet cause stress to the aortic wall and induce ROS (reactive oxygen species) generation and inflammation. ROS and inflammation lead to infiltration of inflammatory cells, cytokine productions, increased MMP (MMP2 and MMP9) activity, and medial vascular smooth muscle cell (VSMC) apoptosis. These result in elastin degradation and VSMC loss, leading to aortic expansion and abdominal aortic aneurysm (AAA) formation. In response to aortic wall stress, HO-1 is induced (+HO-1) in the vessel wall to serve as an endogenous adaptive mechanism to lessen ROS and inflammation. HO-1 induction may also reduce the pathological cellular processes and limit AAA progression. In the absence of HO-1 (-HO-1), aortic stress markedly enhances ROS production and inflammation. Inflammatory infiltrate, inflammatory cytokines, MMP activity, and VSMC apoptosis are also markedly elevated, leading to severe elastin degradation and VSMC loss. These ultimately result in exacerbated response, including AAA and TAA formation, increased incidence and aneurysmal area, and increased severity and rupture rate.

In conclusion, we show that although HO-1 is barely detectable in the aortic wall under basal conditions, it is induced during AAA progression (Figure 10). This induction may provide an endogenous protective mechanism to lessen aortic wall stress-induced ROS and inflammation that lead to inflammatory cell infiltration, cytokine production, MMP activity (MMP2 and MMP9), and VSMC apoptosis (Figure 10). Although our results suggest that medial layer is likely to be the first responder to angiotensin II stimulation by upregulating HO-1 expression and increasing MMP2 activity, infiltrated immune cells also contribute substantially to pathological events by secreting inflammatory cytokines and increasing MMP9 activity. The concerted actions of VSMCs and inflammatory cells result in elastin degradation and medial VSMC loss, ultimately leading to aortic expansion and AAA formation (Figure 10). HO-1 induction may also reduce these pathological cellular processes and limit severity of AAA. Lack of HO-1 markedly enhances oxidative stress, medial VSMC loss, and MMP activity, leading to exacerbated aortic wall degeneration and aneurysm formation including AAA and TAA, and rupture rate (Figure 10). We demonstrate in the present study an essential protective role of HO-1 in the pathogenesis of aortic aneurysm. With the antioxidative and anti-inflammatory properties of HO-1, increasing HO-1 expression in the aorta might be a potentially valuable approach for preventing/treating AAA disease.

## MATERIALS AND METHODS

### Animals

HO-1-deficient (HO-1^−/−^) mice were generated previously [18] and maintained on a 129sv and C57BL/6 mixed genetic background by intercrossing HO-1^+/−^ mice. To generate mice deficient in both HO-1 and apoE, we crossed HO-1 mice with apoE^−/−^ mice (C57BL/6 background, Jackson Laboratory). Genotyping was performed with genomic DNA isolated from tail biopsy by PCR [35]. The mice of HO-1^+/−^apoE^−/−^ genotype (female HO-1^−/−^apoE^−/−^ are infertile) were then intercrossed for more than 10 generations to obtain mice on a homogeneous mixed genetic background of 129sv and C57BL/6. Mice were housed in a specific pathogen-free animal facility at National Health Research Institutes, Taiwan. All experimental procedures were performed in accordance with NIH guidelines (Guide for the care and use of laboratory animals) and approved by the Institutional Animal Care and Use Committee of National Health Research Institutes, Taiwan (#NHRI-IACUC-101144-A).

### Mouse model of angiotensin II-induced abdominal aortic aneurysm formation

Approximately 12-week-old male HO-1^+/+^apoE^−/−^ and HO-1^−/−^apoE^−/−^ mice were subjected to an AAA formation model as described [36, 37]. Mice were anesthetized with isoflurane vapor by inhalation using a Matrx VIP 3000 Vaporizer (Midmark Corp.), 4-5% initially and 1-3% during the procedure. An Alzet model 2004 osmotic minipump (DURECT) filled with saline or angiotensin II (Sigma) was implanted into the subcutaneous space in the back of the neck and angiotensin II was infused at a rate of 1000 ng/kg/min. Following minipump implantation, mice were fed a high fat diet containing 1.25% cholesterol and 20% fat (Research Diet). At indicated time points, mice were sacrificed by an overdose of tribromoethanol solution (500-750 mg/kg) by IP injection, perfused with saline, followed by 10% formalin. The aortas were then carefully dissected, excised, photographs taken, and processed for histological analysis.

The abdominal aorta with or without aneurysm formation was measured and scored according to Daugherty's modified classification [19] none, I, II, III, and rupture: none, no AAA formation; type I, 1.5-2 times of a normal suprarenal aorta; type II, a single large dilation more than 2 times the diameter of a normal suprarenal aorta; type III, multiple dilations extending proximal to the suprarenal region; rupture, death due to rupture of aneurysm. Aneurysm was assessed one-dimensionally by measuring the aneurysmal length along the aorta and expressed as % of aortic length. To quantify aneurysm two-dimensionally, the aortic aneurysmal area (mm^2^) was measured.

### Measurements of blood pressure and plasma cholesterol

A noninvasive tail-cuff method was used to measure systolic blood pressure (SBP) using a non-preheating MK-2000ST system (Muromachi Kikai Corp.). Conscious mice were placed in special mouse holders and acclimated to the device for 10 min before measurement. A minimum of 3 serial measurements was made and the average value calculated. The SBP of each mouse was measured at baseline before angiotensin II infusion and at 4 weeks after infusion. Four weeks after saline or angiotensin II infusion, mice were fasted and blood collected. Plasma total cholesterol levels were then measured using Fuji Dri-Chem Slide TCHO-PIII with a Fuji Dri-Chem Analyzer (Fuji Photo Film Corp.).

### Histological analysis and immunohistochemistry

Vessel sections (4 μm) were stained with H&E for morphology. To assess integrity of elastin layers, we stained sections with Verhoeff's stain (Sigma) for elastin. Three sections at 300-μm intervals were used to determine elastin degradation grade. The elastin degradation was graded [38] as follows: grade 1, no degradation; grade 2, mild degradation; grade 3, severe degradation; grade 4, aortic rupture. To detect HO-1 expression, we performed immunostaining by incubating aortic sections with HO-1 antibody (Enzo, 1:500). HO-1 expression levels were quantified by colorimetric analysis using NIH ImageJ software and expressed as % positive area per section. To identify immune cells, sections were incubated with CD45 antibody (BD Bioscience, 1:500). To assess cytokine expressions in AAA sections, we performed MCP-1 (Abcam, 1:200), IL-6 (Abcam, 1:800), and TNF-α (Sigma, 1:200) immunostaining. To detect VSMCs, sections were stained with SM α-actin antibody (Sigma, 1:8,000). Positive staining areas of SM α-actin were quantified by colorimetric analysis using NIH ImageJ software and expressed as % SM α-actin-positive area per section. To identify infiltrated macrophages, sections were incubated with an antibody against the common macrophage antigen Mac3 (BD Bioscience, 1:500). To assess macrophage accumulation, the number of Mac3-positive cells (in the media and adventitia) in three sets of sections at 300-μm intervals was counted and averaged, and expressed as positive cell number per section. To detect apoptotic cells, cleaved-capase3 staining (Cell Signaling Technology, 1:500) was performed. The number of cleaved-caspase3-positive cells in the medial layer from three sets of sections at 300-μm intervals was counted, averaged, and expressed as medial apoptotic cell number per section.

### Measurement of reactive oxygen species levels

To assess aortic oxidative stress levels, mice were perfused with cold PBS for 5 min, abdominal aortas isolated, embedded in OCT compound (Leica), and frozen immediately. To measure production of ROS in the aneurysmal aortic wall, freshly prepared frozen aortic sections (10 μm) were incubated with 5 μmol/L fluorescent dye dihydroethidium (DHE, Molecular Probes) at 37°C for 30 min in a humidified chamber and protected from light. Digital images were captured by Olympus microscope system (BX51, Japan), and the red fluorescence intensity was quantified by using NIH ImageJ software. The fluorescence intensity was expressed relative to that of HO-1^+/+^apoE^−/−^ mice (set as 1) at each time point.

### Matrix metalloproteinase activity assays

To measure aortic MMP activity, we performed *in situ* zymography using EnzChek^®^ gelatinase/collagenase assay kit (Molecular Probes). We incubated freshly cut frozen aortic sections (10 μm) with a fluorogenic gelatin substrate (DQ gelatin) at 37°C for 90 min in a humidified chamber and protected from light according to manufacturer's protocol. Proteolytic activity was detected as green fluorescence. For negative controls, we preincubated sections with gelatinase/collagenase inhibitors (Molecular Probes) for 30 min before adding substrate, and no detectable gelatinolytic activity was observed. Digital images were captured by Olympus microscope system (BX51). The green fluorescence intensity was quantified by using NIH ImageJ software and expressed as fold of HO-1^+/+^apoE^−/−^ mice at each time point. For *in vitro* MMP activity, conditioned medium from VSMCs or peritoneal macrophages was collected and concentrated 50-fold with an Amicon^®^ Ultra-15 centrifugal filter (Millipore). An aliquot of the concentrated medium was then subjected to SDS-PAGE zymography essentially as described [39]. In brief, conditioned medium was electrophoresed in 8% gels containing 0.8 mg/mL gelatin (Sigma). The gels were subsequently incubated with renaturing buffer (2.7% Triton X-100) for a total of 45 min (15 min x 3 times) followed by zymography developing buffer (50 mmol/L Tris, 0.2 mol/L HCl, 5 mmol/L CaCl_2_, 0.02% Brij35) at 37°C for 40 h. The gels were then stained with PhastGel^™^ Blue R (GE Healthcare).

### Vascular smooth muscle cell culture, viability, MMP2 activity, and protein expressions

Approximately 8 weeks old HO-1^+/+^apoE^−/−^ and HO-1^−/−^apoE^−/−^ mice were sacrificed by an overdose of tribromoethanol solution (500-750 mg/kg) by IP injection. Primary VSMCs were then isolated from aortas and cultured in DMEM as described [40]. Cells of passages 3-6 were used for experiments. To determine the effect of oxidant on cell viability, HO-1^+/+^apoE^−/−^ and HO-1^−/−^apoE^−/−^ VSMCs were plated in 24-well plates (4×10^4^ cells/well), serum starved in quiescent medium (0.2% FBS) for 36 h, then stimulated with increasing concentrations of H_2_O_2_ for 24 h. MTT assays were then performed and cell viability presented as percentage of control without H_2_O_2_. To evaluate the effect of angiotensin II on MMP2 activity, HO-1^+/+^apoE^−/−^ and HO-1^−/−^apoE^−/−^ VSMCs were serum starved, stimulated without or with different concentrations of angiotensin II for 24 h, and the conditioned medium collected. Conditioned medium was then concentrated and subjected to zymography to determine MMP2 activity. Total proteins were isolated from VSMCs for Western blotting. To determine MMP2 and HO-1 protein expressions, the blots were incubated with MMP2 (Abcam, 1:500) and HO-1 (Enzo, 1:500) antibodies, respectively. The blots were subsequently probed with a pan-actin antibody (Millipore, 1:80,000) to verify equivalent loading.

### Peritoneal macrophage preparation, MMP9 activity, and protein expressions

Primary peritoneal macrophages were harvested from HO-1^+/+^apoE^−/−^ and HO-1^−/−^apoE^−/−^ mice according to the method described previously [41]. Briefly, 4% Brewer thioglycollate (BD Bioscience) medium was injected into the peritoneal cavity of 6-8 weeks old mice. Four days after injection, mice were euthanized by CO_2_ inhalation. Primary peritoneal macrophages were harvested and plated on culture dishes with RPMI 1640 medium containing 10% FBS. After 2 h incubation, nonadherent cells were removed by washing with RPMI medium. Adherent cells were then used for experiments. To determine the effect of different mediators on MMP9 activity, macrophages were treated with 10 μmol/L angiotensin II or 100 ng/mL eotaxin (ProSpec). At different time points following stimulation, conditioned medium was collected for measuring MMP9 activity and cytokine productions. Total proteins were prepared from macrophages for Western blot analysis to detect MMP9 (Abcam, 1:1,000) and HO-1 (Enzo, 1:500). Equivalent loading was verified by incubating blots with a pan-actin antibody (Millipore, 1:80,000).

### Cytokine and chemokine analysis

The concentration of inflammatory cytokine MCP-1, IL-6, and TNF-α in the conditioned medium of macrophages was determined using an ELISA kit (eBioscience) in triplicate following manufacturer's instructions. The cytokine concentration was then normalized to total protein amount and expressed as pg/μg protein. To perform rescue experiments, HO-1^−/−^apoE^−/−^ macrophages were treated with 0, 2 or 5 μmol/L of CO-releasing molecule CORM-3 (Sigma) in the presence or absence of 10 μmol/L angiotensin II for 24 h. Conditioned medium was then collected for inflammatory cytokine MCP-1, IL-6, and TNF-α measurements and presented as fold of control without angiotensin II and CORM-3 treatment. To evaluate mouse circulating eotaxin levels at different time points after AAA induction, we collected blood samples from HO-1^+/+^apoE^−/−^ and HO-1^−/−^apoE^−/−^ mice 2, 3, and 4 weeks following angiotensin II infusion. To avoid interference of lipids on chemokine measurement, lipids were removed from plasma using PHM-L LIPOSORB Absorbent (Millipore). Plasma eotaxin concentration was then measured in triplicate using a mouse eotaxin immunoassay kit (R&D Systems) and expressed as pg/mL plasma.

### Statistical analysis

Data are presented as mean ± S.E. of at least three independent experiments. SBP data were compared by paired Student's *t*-test. Others were analyzed by unpaired Student's *t*-test. Differences in thrombus formation or TAA+AAA formation between the two genotypes of mice were analyzed by Fisher's exact test. Statistical significance is considered at *P* value < 0.05.
